# Integrating binding and expression data to predict transcription factors combined function

**DOI:** 10.1186/s12864-020-06977-1

**Published:** 2020-09-07

**Authors:** Mahmoud Ahmed, Do Sik Min, Deok Ryong Kim

**Affiliations:** 1 Department of Biochemistry and Convergence Medical Sciences and Institute of Health Sciences, Gyeongsang National University School of Medicine, Jinju, 52727 Republic of Korea; 2 College of Pharmacy, Yonsei University, Incheon, 21983 Republic of Korea

**Keywords:** BETA, DNA-binding, Cooperative-binding, Competitive-binding, Transcription-factor, R-package, YY1

## Abstract

**Background:**

Transcription factor binding to the regulatory region of a gene induces or represses its gene expression. Transcription factors share their binding sites with other factors, co-factors and/or DNA-binding proteins. These proteins form complexes which bind to the DNA as one-units. The binding of two factors to a shared site does not always lead to a functional interaction.

**Results:**

We propose a method to predict the combined functions of two factors using comparable binding and expression data (target). We based this method on binding and expression target analysis (BETA), which we re-implemented in R and extended for this purpose. target ranks the factor’s targets by importance and predicts the dominant type of interaction between two transcription factors. We applied the method to simulated and real datasets of transcription factor-binding sites and gene expression under perturbation of factors. We found that Yin Yang 1 transcription factor (YY1) and YY2 have antagonistic and independent regulatory targets in HeLa cells, but they may cooperate on a few shared targets.

**Conclusion:**

We developed an R package and a web application to integrate binding (ChIP-seq) and expression (microarrays or RNA-seq) data to determine the cooperative or competitive combined function of two transcription factors.

## Background

### Motivation

The binding of a transcription factor to the regulatory region (e.g. gene promoter or enhancer) of a particular gene induces or represses its gene expression [1]. High-throughput chromatin immunoprecipitation (ChIP) experiments identify hundreds or thousands of binding sites for most factors [2]. Therefore, methods are needed to determine which of these sites are true targets and whether they are functional [3]. Perturbing the transcription factor coding gene by overexpression or knockdown and measuring the effects on cellular gene expression provides useful information on the function of the factor [4]. Methods exist to integrate binding and gene expression data of the factor perturbation to predict the direct target regions (e.g. genes) [5, 6].

Transcription factors share their binding sites with other factors, co-factors and/or DNA-binding proteins [7, 8]. These transcriptional proteins form one-unit complexes which bind to the regulatory regions. Moreover, the binding of a protein to a specific region of the DNA can modulate the binding of other proteins elsewhere [7]. In the former case, the binding site of two or more factors can be determined by pulling down the areas of the chromatin bound to the factors individually and calculating the overlapping ChIP peaks (binding sites). Alternatively, re-ChIP experiments can be used to the same effect [9]. Perturbing the factors individually in comparable experiments by overexpression or knockdown helps identify their functional effects on gene expression.

### Methods for data integration and target prediction

Methods for predicting direct gene targets vary depending on the type of data they use. Some methods use a single data source such as regulatory sequences, chromatin accessibility, ChIP peaks or transcriptomics data [10, 11]. coTRaCTE uses DNase I hypersensitive site (DHS-Seq) data to identify co-binding of pairs of transcription factors [12]. Methods that combine more than one type of data also exist. ChIP-Array considers the binding-enrichment of a factor from ChIP and the differential expression under factor perturbation [13]. EMBER uses a machine learning algorithm to detect targets from the same types of data [14].

By making simple assumptions about transcription factor binding and effects on gene expression, the combined functions of two or more factors can be inferred. Ouyang et al. constructed an association signal matrix for multiple transcription factors based on the distances between their binding sites and the transcription start sites (TSS) [15]. The matrix is normalized and scaled then subjected to principal component analysis which is used to predict the log-transformed gene expression under factor perturbation. The explanatory components are used as weights to approximate interactions between the different factors. Binding and expression target analysis (BETA) integrates the binding and expression data to predict direct targets [6]. Genes with binding peaks and whose expression is changed by factor perturbation are ranked higher in importance.

Several R/Bioconductor packages exist for the purpose of identifying transcription factor gene targets and for integrating binding and expression data in general. Although these packages do not always have the same goal, they attempt to integrate ChIP and expression data. rTRM attempts to identify the transcriptional regulatory modules (TRMs), which are complexes of transcription factors and co-factors by integrating ChIP-seq, gene expression and protein-protein interaction data [16]. The TFEA.ChIP package curates large quantities of data from different sources and uses this data to build a model or a database to query for targets [17]. Finally, transcriptR integrates ChIP- and RNA-Seq data for an entirely different purpose [18]. It uses the ChIP data to *denovo* identify transcripts which are then used to map the reads from the RNA-Seq data to quantify gene expression.

## Implementation

### Proposing target analysis

**Fig. 1:**
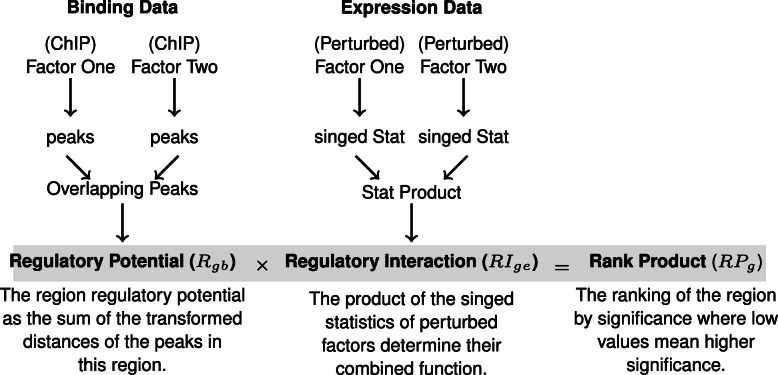
Integrating binding and expression data to predict the combined function of two transcription factors. The binding peaks from ChIP experiments of two factors are used to find their shared binding sites in the regions of interest. The distances between the peaks and the regions are used to calculate peak scores. The sum of the scores of all peaks assigned to a region is its regulatory potential. The product of signed statistics from gene expression profiling of the factors perturbation is used to determine the magnitude and the direction of their regulatory interactions. The products of the ranks of the regulatory potential and the regulatory interactions are used to rank the regions of interest

To determine the functional interaction of two transcription factors, we first identify their shared binding sites and the effects on gene expression of perturbing each separately. Two factors work cooperatively when they share a binding site and when they both induce or repress the gene [7]. By contrast, two factors compete on a specific site when the binding of either has opposite effects on the target gene expression [19]. Figure 1 summarizes the proposed method. One advantage of our approach is that it assigns numerical values to each target which can be used to obtain ranked predictions. Another is that the predicted combined function (interaction) of the two factors is easily interpreted as compared to classification trees or amounts of variance proposed by other methods.

Here, we summarize the formulation of BETA [6]. Then, we describe extending this method for the purpose of integrating comparable datasets on two factors to predict their combined function. We provide a fast and flexible implementation of this approach in an R package called target and an accompanying Shiny interactive application [20, 21]. Finally, we introduce examples from simulated and real data to evaluate the new method.

### Binding and expression target analysis (BETA)

The BETA algorithm is composed of five steps [6]:

- Select the peaks (*p*) within a specified range in a region of interest (*g*) which could be a promoter region.
- Calculate the distance (*Δ*) between the center of each peak and the start of the region expressed relative to the range in kb.
- Calculate the score of each peak (*S*_*p*_) as the transformed exponential of the distance, *Δ*. These parameters were chosen to derive a monotonically decreasing function that approximates the empirical data [22], as follows:
  1 $$  S_{p} = e^{-\left(0.5+4\Delta\right)}  $$
- Calculate the region’s regulatory potential (*S*_*g*_) as the sum of all peaks scores (*S*_*p*_) [22], as follows:
  2 $$  S_{g} = \sum_{i=1}^{k} S_{pi}  $$
  where *p* is {1,...,*k*} peaks within the region of interest.
- Rank all regions based on their regulatory potential, *S*_*g*_, to give (*R*_*gb*_) and based on their differential expression (fold-change or t-statistics) from the factor perturbation experiment to give (*R*_*ge*_). The products of the two ranks (*RP*_*g*_) predict direct targets.
  3 $$  RP_{g} = \frac{R_{gb}\times R_{ge}}{n^{2}}  $$
  where *n* is the number of regions *g*.

### Regulatory interaction (RI) term for predicting combined functions

To determine the relationship of two factors *x* and *y* on a region of interest where they have common peaks, we define a new term, the regulatory interaction (*RI*), as the product of two signed statistics from comparable perturbation experiments. The ranks of the new term (*RI*_*ge*_) and the previously defined regulatory potential (*R*_*gb*_) are then multiplied.

4 $$  RI_{g} = x_{ge}\times y_{ge} \quad\text{and}\quad RP_{g} = \frac{R_{gb}\times RI_{ge}}{n^{2}}  $$

This term would represent the interaction magnitude assuming a linear relation between the two factors. The sign of the term would define the direction of the interaction where positive means cooperative and negative means competitive. To determine the combined function of two factors, the targets are first divided into groups based on the regulatory interactions cutoffs or quantiles. For example, regions with positive interaction would represent regions of cooperation and vice versa. Then the empirical cumulative distribution function (ECDF) of the regulatory potentials of the regions is calculated separately for each group. The ECDFs approximate the aggregate potentials of the groups, which are compared to each other. If the curve of one group lies above that of the other, the regions in the first group have higher regulatory potentials and hence represent the dominant interaction type.

### Testing the difference between the aggregate functions

The curves of the aggregate functions in each group can be visually inspected for differences. To formalize the comparison, the Kolmogorov-Smirnov (KS) test is used, as suggested in the original BETA paper [5]. Two samples KS tests whether the distribution of the functions of two groups were drawn from the same distribution. In particular, the differences in shape and location between two curves are tested. The larger the distance or the side-shift between the two functions, the larger the difference in the factor aggregate functions. This test is applied using ks.test from R base [23].

### The target R package

We developed an open source R package (target) to implement BETA and extend it to apply to factor combinations (https://bioconductor.org/packages/target/). The package leverages the Bioconductor data structures such as GRanges and DataFrame to provide fast and flexible computation [24]. Similar to the original python implementation, the input data are the identified peaks from the ChIP-Seq experiments and the expression data from RNA-Seq or microarray perturbation experiments. The final outputs are associated peaks and direct targets. The first is the filtered peaks each assigned to one of the specified regions. The second is the predicted targets of the factors ranked by importance.

We use the term peaks to refer to the GRanges object that contains the coordinates of the peaks. Similarly, we use the term regions to refer to a similar object that contains information on the regions of interest: genes, transcripts, promoter regions, etc. In both cases, additional information on the ranges can be added to the object as metadata. Table 1 lists the functions in the R package along with each ones’s specific description, input and output. The first five functions correspond to the five steps of the algorithm presented earlier (not intended to be used directly). The rest of the functions corresponds to the final outputs.

**Table 1:** Functions in the target R package

Function	Description	Input	Output
merge_ranges	Merge overlapping peaks & regions.	peaks & regions	Merged ranges
find_distance	Calculate the distance between the centers of peaks & regions.	peaks & regions	Distances
score_peaks	Calculate regulatory scores for peaks in relation to regions.	Distances	Peak scores
score_regions	Calculate regulatory scores for regions.	Peak scores & region IDs	Regions scores
rank_product	Rank regions based on the regulatory potential & expression statistics.	Regions scores, expression statistics & region IDs	Regions rank products
associated_peaks	Select overlapping peaks & regions & calculate a score for each peak in relation to a region.	peaks & regions	Assigned peaks
direct_target	Select & rank regions with overlapping peaks.	peaks & regions	Assigned targets
plot_predictions	Plot the ECDF of the regions’ ranks by group.	Ranks & group factor	ECDF plot
test_predictions	Test the ECDF of the ranks in the regions in each group are from different distribution.	Ranks & group factor	t-statistics & *p*-values

### The web interface

The target package comes with an interactive user interface that can be used to perform the same predictions. The interface is also available as a web application (https://mahshaaban.shinyapps.io/target-app/). The inputs to the web application are slightly different. Instead of GRanges objects, users provide the input in text format. The binding data can be in standard bed format. The expression data can be in a tab separated text file with at least three columns (region names and statistics columns for each factor). Finally, the user has the option to choose a reference genome from the built-in database or upload a custom genome file in standard bed format. One column in this file should be identical to the names column in the expression data, since the two are merged at some point to select the peaks belonging to each region. The output from the web application is similar to that of the package. The tables of associated peaks and direct targets are calculated automatically. The predictions can be summarized using plots and tested for statistical significance. The output can be downloaded for further analysis.

### Availability

target is available as an open source R/Bioconductor package (https://bioconductor.org/packages/target/). The accompanying interactive application can be invoked locally through R or accessed directly on the web (https://mahshaaban.shinyapps.io/target-app/). The source code for the package and the interactive application is available at (https://github.com/MahShaaban/target) under the GPL-3 license.

## Results

### Simulation of cooperative and competitive binding factors

**Fig. 2:**
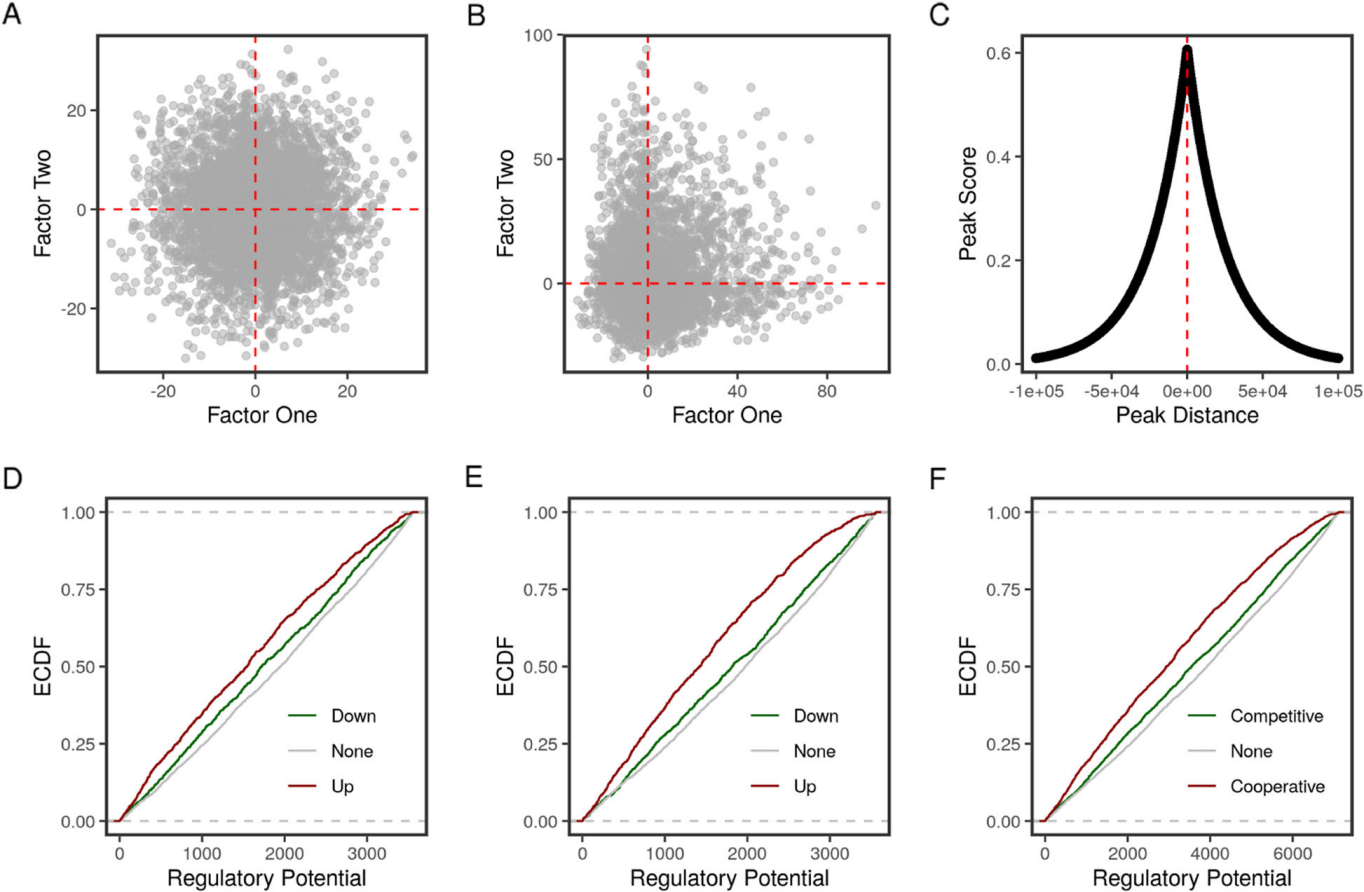
Simulation of cooperative binding of two factors. **a** Scatter plot of the two randomly simulated statistics. **b** Scatter plot of the two simulated statistics after introducing parallel (positive) changes to the second factor. **c** Plot of the peak scores vs their distance from the overlapping regions. **d** Predicted function of factor one. **e** Predicted function of factor two. **f** Predicted combined function of the two factors. Empirical cumulative distribution functions (ECDF) of the regulatory potential/interactions are calculated separately for the groups of regulated regions

**Fig. 3:**
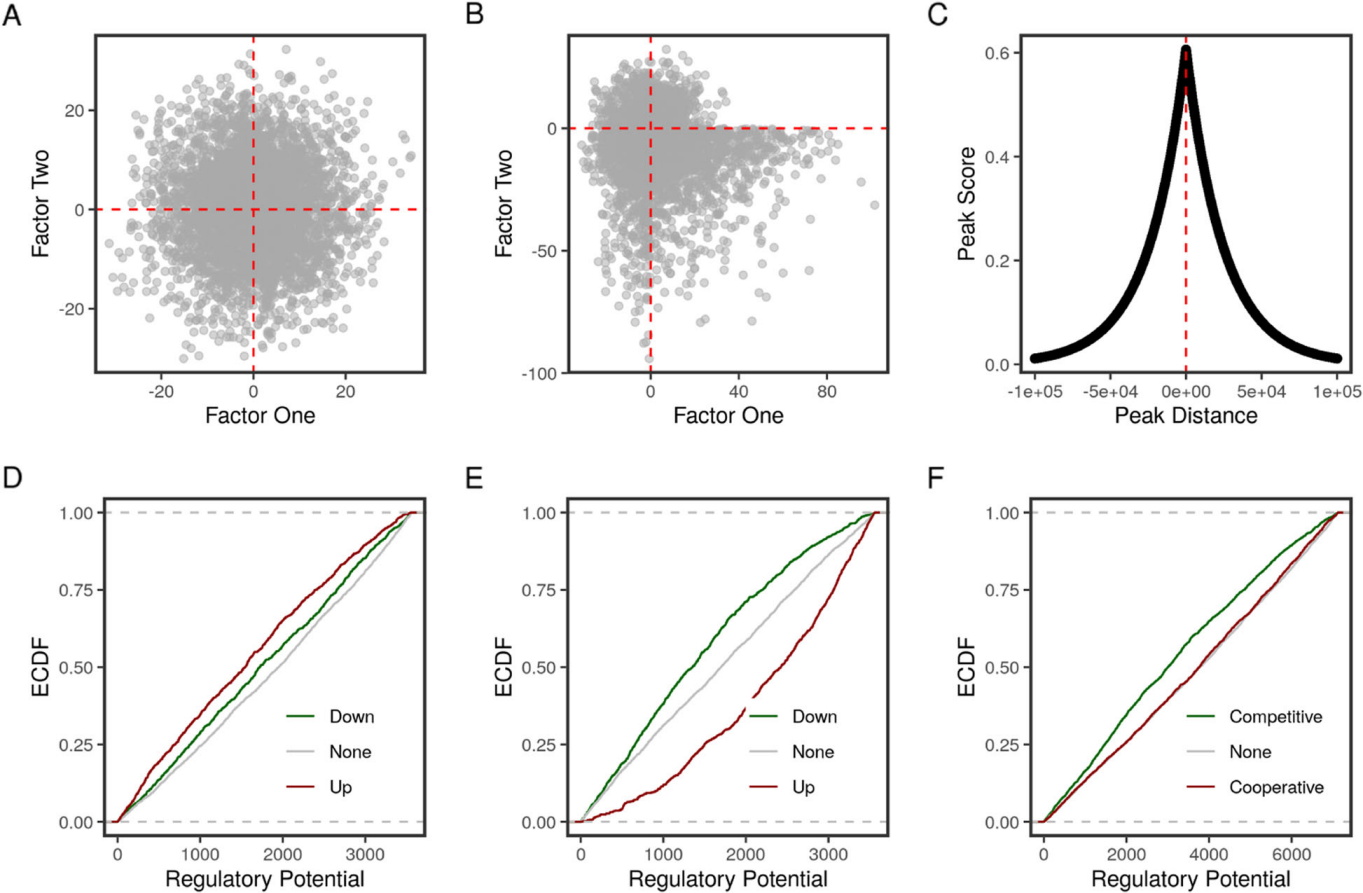
Simulation of competitive binding of two factors. **a** Scatter plot of the two randomly simulated statistics. **b** Scatter plot of the two simulated statistics after introducing opposing (negative) changes to the second factor. **c** Plot of the peak scores vs their distance from the overlapping regions. **d** Predicted function of factor one. **e** Predicted function of factor two. **f** Predicted combined function of the two factor. Empirical cumulative distribution functions (ECDF) of the regulatory potential/interactions are calculated separately for the groups of regulated regions

The target package contains two simulated datasets. sim_peaks contains randomly generated peaks with random distances from the transcription start sites (TSS) on chromosome 1 of the mm10 mouse genome. sim_transcripts is random values to simulate statistics on transcript expression as a consequence of perturbing two factors. To illustrate how the proposed method detects cooperative or competitive binding conditions, we introduced a bias to the singed statistics of a 1000 transcripts. In the case of cooperative factors, we multiplied the values corresponding to one of the two factors by 3. This magnified the effect of the factor perturbation in the same direction (Fig. 2). Multiplying the signed statistics of one of the two factors by -3, on the other hand, reversed the sign and gave a pattern of functionally competing factors (Fig. 3).

Before making any changes, the statistics of the two factors are completely random (Fig. 2a). Multiplying the statistics of the second factor by 3 in transcripts with nearby peaks skewed the scatter of the two factors in the positive direction (Fig. 2b). As expected, the peak score was an exponentially decreasing function of the distance between the peaks and the TSSs (Fig. 2c). Applying the standard target analysis to the two factors individually showed a higher proportion of induced/up-regulated targets with higher regulatory potential (Fig. 2d & e). When including the statistics of the two factors in the calculations, the regulatory interaction (RI) was used to rank the targets. As expected, the curve of the targets with positive regulatory interaction shifted upwards (Fig. 2f).

By contrast, the induced negative change to the statistics of factor two skewed the scatter to the bottom quadrants as compared with a random distribution (Fig. 3a)_. The target analysis of the individual factors showed opposite patterns, factor one was inducing/up-regulating targets and factor two was repressing/down-regulating targets (Fig. 3d & e). Finally, the combined function of the two factors was competitive in nature as they exerted opposing effects on their common targets (Fig. 3f).

### YY1 and YY2 cooperate on their shared gene targets in HeLa cells

Yin Yang 1 transcription factor (YY1) and YY2 belong to the GLI-Kruppel family of zinc finger transcription factors, which are involved in repressing and activating a diverse set of genes [25, 26]. We used binding and expression data on YY1 and YY2 in HeLa cells to predict the effects of the two factors on specific and shared gene targets. Two ChIP-Seq datasets were prepared using a ChIP antibody against either YY1 (GSE31417) or YY2 (GSE96878) in HeLa cells [27, 28]. Raw reads were mapped to the human genome (hg19) using BOWTIE2 and peaks were called using MACS2 [29, 30]. The processed data was obtained in the form of narrow peaks from the ChIP-Atlas database [31]. Expression profiling using microarrays of the transcription factors knockdown was performed in HeLa cells (GSE14964) [32]. Probe intensities were log2 transformed and the fold-changes of knockdown vs. control were calculated using LIMMA [33]. The processed data was obtained in the form of differential expression in the factor knockdown condition vs control from the KnockTF database [34].

**Fig. 4:**
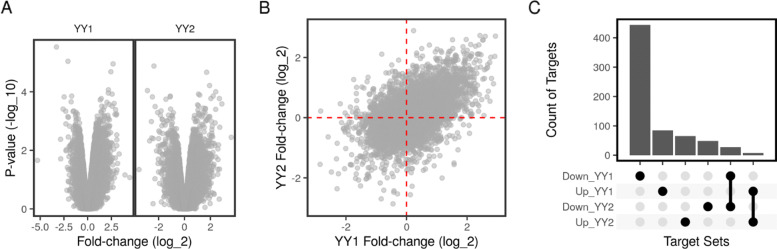
Differential expression of YY1 and YY2 in knockdown vs control HeLa cells. Probe intensities from microarrays of YY1 or YY2 (n = 3) knockdown and control cells (n = 3) were aggregated by gene and used to perform differential expression analysis (GSE14964). Gene expression in the YY1- and YY2-knockdown samples was compared to that of the control samples individually. **a** Volcano plots show the fold-change (log_2_) and *p*-values (-log _1_0) in each comparison. **b** The fold-change (log_2_) of the YY1- and YY2-knockdown samples are shown as a scatter plot. **c** The count of regulated (Up/Down) genes in by YY1 or YY2-knockdown and their intersections are shown as bars

Knockdown of YY1 in HeLa cells had a larger effect on the gene expression than did knockdown of YY2 (Fig. 4a). This was also reflected in the larger number of binding peaks of YY1 near the TSSs. Specifically, YY1 knockdown resulted in the down-regulation of a large number of genes while the YY2 knockdown had the opposite effect but on a smaller number of genes. However, the overall fold-change in the knockdown of either factors was well correlated (Fig. 4b), suggesting that the effects of the two factors on the shared targets may be different than the effects of each on its specific targets. Indeed, the number of potential targets of each factor exceeded the number of shared targets (Fig. 4c).

**Fig. 5:**
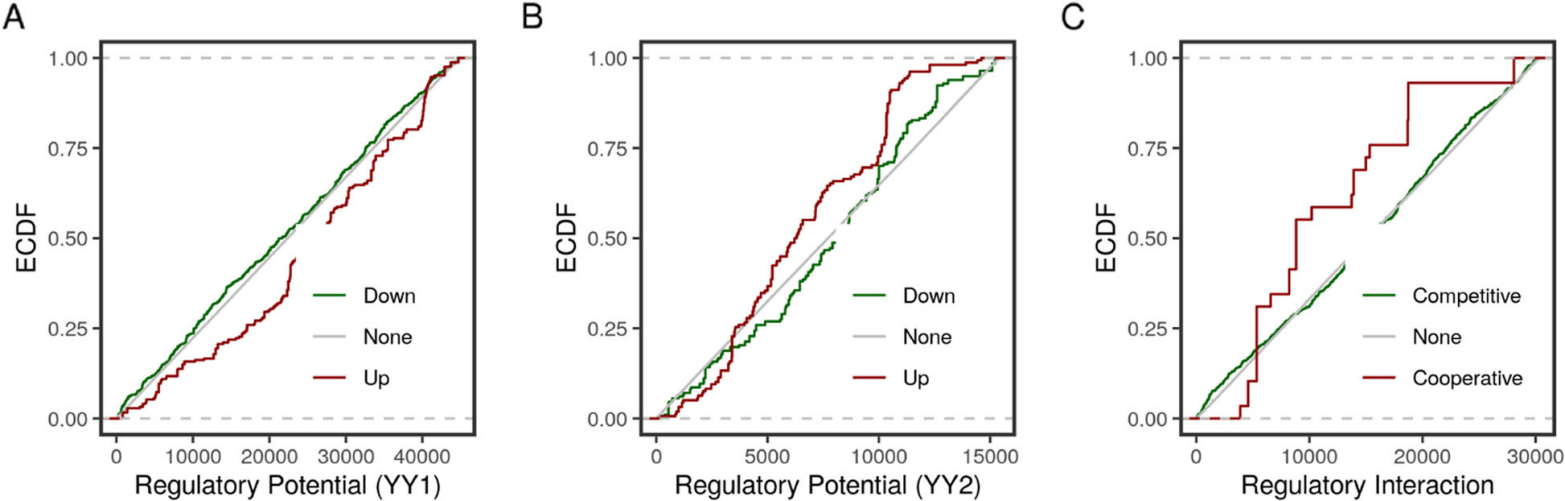
Predicted functions of YY1 and YY2 on specific and shared targets in HeLa cells. The target analysis was applied using two sets of data from the HeLa cells: expression data in YY1- and YY2-knockdown cells (GSE14964) and two sets of ChIP peaks using antibodies for YY1 (GSE31417) and YY2 (GSE96878). Predicted targets were ranked based on the distance of their peaks to the transcription start sites (TSS) and their fold-change. The empirical cumulative distribution function (ECDF) of the regulatory potential of each group of targets (Down, None or Up-regulated genes) of **a** YY1 and **b** YY2 was calculated. **c** The shared targets were ranked based on the distance of their peaks to the TSS in which they had overlapping peaks and the product of the corresponding fold-changes. The ECDF of the regulatory potential of each group of targets (Competitively, None or Cooperatively regulated genes) was calculated

Individually, the two factors had overall opposing functions on a larger number of targets. However, on the shared set of targets, the two transcription factors may cooperate. YY1 knockdown down-regulated many targets, and these had higher regulatory potentials (Fig. 5a & Table 2). Conversely, the overall effect of the YY2 knockdown was positive on the highly-ranked targets (Fig. 5b & Table 2). Considering only the shared targets of both factors, the combined effect of the knockdown of the two factors was positive. That is, binding of the two factors on a shared target site may cooperatively induced or repressed gene expression, with a few but strong exceptions (Fig. 5c & Table 2).

**Table 2:** Testing YY1 and YY2 combined functions

Factor	Test	Statistic	*P*-Value
YY1	Down vs Up	0.79	0e+00
YY2	Up vs Down	0.41	5e-13
Two Factors	Cooperate vs Compete	0.97	0e+00

The implication of these observations is that YY1 and YY2, despite being members of the same family, each has unique targets. Since the knockdown of a transcription factor reverses its functional role, YY1 likely induces more target genes than it represses. The opposite would be true for YY2, albeit with fewer targets. Finally, on the smaller set of common targets, for which the two factors share binding sites, they are expected to positively cooperate in induction of target gene expression. This may not be the case for a few strong shared targets where the two factors have opposing effects. These findings agree with previous studies in affirming the antagonistic roles of YY1 and YY2 [32]. On their strongest targets the two factors may compete, but we also suggest a less appreciated cooperative function of the two factors.

## Discussion

In this article, we provide a fast and flexible implementation of the BETA algorithm for predicting direct targets of transcription factors and chromatin remodelers from binding and expression data. We extended this method to determine the combined function of two factors that bind to the same region. The overlapping binding sites of the two factors are used to calculate the regulatory potential of the factors on the regions of interest. The signed statistics of the perturbation of the factors in comparable experiments are used to calculate a regulatory interaction which determines their combined function; cooperative or competitive. We developed an R package and a web application to apply these two methods.

The proposed method requires experimental data with a specific design. Comparable sets of data for the two factors are required: binding data using ChIP and gene expression data under factors perturbation (overexpression or knockdown). Therefore, the practical application of this method is limited by the availability of certain types of data. It is not possible to adapt the method to work with predicted binding sites instead of ChIP peaks, since the regulatory potential of a factor is a function of the distances of its peaks from the regulatory region of the targets. It is not clear whether binding sites predicted by other methods can be assigned numeric values that follow the same function shape or distribution.

Several modes of regulatory interaction are not captured by the method, in particular non-linear interactions and assisted binding. In the latter case, the binding of one factor increases or decreases the binding affinity of another at a different site. Since the starting point of this method is identifying the overlapping binding peaks of the two factors, this form of interaction would not be recognized. Finally, it is not possible to distinguish the binding of one protein to another from direct DNA binding. In either cases, the interpretation of the regulatory interaction would be identical since the binding peaks would be predicted all the same.

## Conclusion

In this article, we present a method for identifying the combined functions of two transcription factors or DNA binding proteins. This method integrates binding (ChIP-seq) and expression (microarrays or RNA-seq) data to determine the cooperative or competitive combined function of the factors. We implemented this method in an R package and a web application.

## Availability and requirements

- Project name: target
- Project home page: https://bioconductor.org/packages/target/
- Operating system(s): Platform independent
- Programming language: R
- Other requirements: R (>= 3.6), BiocGenerics, GenomicRanges, IRanges, matrixStats, methods, stats, graphics and shiny (R packages)
- License: GPL-3
- Any restrictions to use by non-academics: Non

## Data Availability

The datasets analysed during the current study are available in the gene expression omnibus (GEO) repository under the accession numbers GSE31417, GSE96878 and GSE14964.

## Abbreviations

BETA: Binding and expression target analysis
ChIP: Chromatin immunoprecipitation
DHS-Seq: DNase I hypersensitive site
ECDF: Empirical cumulative distribution function
KS: Kolmogorov-Smirnov
TRMs: Transcriptional regulatory modules
TSS: Transcription start sites
YY1: Yin Yang 1 transcription factor
YY2: Yin Yang 2 transcription factor
